# Functional Stratification and Long-Term Care Service Patterns among Discharged Patients with Cancer: A Retrospective Study from Taiwan

**DOI:** 10.7150/jca.134200

**Published:** 2026-05-11

**Authors:** Hui-Wen Po, Ying-Chien Chu, Hui-Chen Tsai, Tzu-Yu Huang, Chung-Yu Chen

**Affiliations:** 1 Department of Nursing, National Taiwan University Hospital Yunlin Branch, Yunlin County, Taiwan.; 2 Department of Internal Medicine, College of Medicine, National Cheng Kung University, Tainan, Taiwan.

**Keywords:** cancer, discharge planning, long-term care, functional status, transitional care, supportive care

## Abstract

**Background:**

As cancer survival improves, a growing number of patients experience functional decline and require supportive care and long-term care after hospitalization. However, the relationship between functional severity and post-discharge long-term care utilization in patients with cancer remains insufficiently characterized. This study examined long-term care service patterns across functional severity levels, as classified by a Case-Mix System, among hospitalized patients with cancer undergoing discharge planning.

**Methods:**

We conducted a retrospective single-center study of adult patients with cancer who received structured discharge planning at a tertiary hospital in Taiwan between January and December 2024. Discharge outcomes and applications for government-funded long-term care services were described for the full cohort. Exploratory multivariable analyses were restricted to patients who applied for long-term care and had complete Case-Mix System assessment data. Modified Poisson regression with robust variance was used for binary service outcomes, Poisson regression for the number of requested service types, and log-linear regression for length of stay.

**Results:**

Among 207 hospitalized patients with cancer who received discharge planning, 141 (68.1%) returned home, 26 (12.6%) were transferred to long-term care facilities, 1 (0.5%) was transferred to another hospital, and 39 (18.8%) died during hospitalization. Of 167 patients discharged home or to care facilities, 75 (44.9%) applied for long-term care services. Among applicants, 19 (25.3%) had mild disability, 36 (48.0%) moderate disability, and 20 (26.7%) severe disability according to Case-Mix System categories. Long-term care utilization showed a non-linear pattern across functional strata. After adjustment for age, sex, and length of stay, moderate disability was associated with a higher number of requested service types than mild disability (adjusted relative risk [aRR] 1.47, 95% CI 1.16-1.87; p = 0.001). Severe disability was associated with greater respite care use (aRR 2.40, 95% CI 1.40-4.09; p = 0.001), lower home-care use (aRR 0.40, 95% CI 0.17-0.95; p = 0.038), and longer hospitalization (ratio 2.82, 95% CI 1.71-4.65; p < 0.001).

**Conclusions:**

Functional stratification using a Case-Mix System was associated with distinct long-term care service patterns among discharged patients with cancer. Moderate disability was linked to broader multi-service needs, whereas severe disability was more strongly associated with respite-oriented care and prolonged hospitalization. In this single-center exploratory cohort, these findings support further validation of functional stratification approaches for post-discharge cancer care transitions.

## Background

Advances in cancer diagnosis, screening, and treatment have transformed cancer from an acute, often fatal illness into a chronic condition, contributing to rising survival rates and longer lifespans 1, 2. This shift has created a growing population of survivors facing long-term complications that extend beyond active treatment, including fatigue, cognitive decline, emotional distress, and reduced physical function 3-5. Older adults, in particular, encounter heightened long-term care (LTC) needs because of comorbidity, polypharmacy, and functional limitation 6, 7.

Despite these increasing demands, healthcare systems remain insufficiently prepared to provide comprehensive post-acute support. Most resources and insurance coverage prioritize acute treatment while underemphasizing home care, rehabilitation, caregiver support, and community-based symptom management 1-3. As post-treatment and post-discharge cancer care becomes more complex, there is a need for pragmatic multidimensional assessment tools that can be embedded in real-world discharge planning. Although tools such as the Geriatric 8 and predictive models can identify vulnerability 7, 8, the Taiwan LTC Case-Mix System (CMS) is already used operationally in discharge-linkage programs to translate functional assessment into service planning 9, 10.

To address this gap, the present study adopted the Taiwan-implemented CMS to evaluate post-discharge LTC needs among hospitalized cancer patients. CMS was chosen because it was the assessment already embedded in our hospital discharge-planning workflow, allowing direct linkage between bedside functional assessment and LTC care planning. The novelty of this study lies in examining whether this real-world functional stratification identifies distinct service packages among hospitalized patients with cancer, rather than simply greater overall utilization. We therefore sought not only to describe the frequency of LTC application after discharge, but also to determine whether CMS strata identify distinct service packages, caregiving contexts, and care complexity. Exploratory multivariable analyses were added to test whether the observed service-utilization patterns remained robust after adjustment for key covariates.

## Materials and Methods

### Study design and participants

This retrospective study analyzed discharge-planning outcomes for adult cancer patients managed at National Taiwan University Hospital, Yunlin Branch, between January and December 2024. Eligible participants were patients aged 18 years or older with a confirmed diagnosis of solid tumor or hematologic malignancy who completed acute inpatient care and received structured discharge-planning services. Patients discharged against medical advice or lacking critical CMS data were excluded from subgroup analyses. The study was approved by the Institutional Review Board of National Taiwan University Hospital (IRB No. 202505089RIND).

### Data sources and variables

Patient data were compiled from the hospital electronic medical record system and the LTC application database maintained by the hospital case-management center. The full discharge-planning cohort comprised 207 patients. For LTC-application analyses, the denominator was restricted to the 167 patients discharged home or to care facilities; patients who died during hospitalization or were transferred to another acute hospital were excluded from the post-discharge LTC-application denominator.

Available variables included age, sex, living arrangement, caregiver structure, disability certificate status, fall history, Activities of Daily Living (ADL), Instrumental Activities of Daily Living (IADL), length of stay, discharge disposition, CMS category, requested LTC services, and service approval status. CMS categories were grouped a priori as mild disability (levels 2-3), moderate disability (levels 4-6), and severe disability (levels 7-8). These three bands were chosen to reflect clinically interpretable low, intermediate, and high dependency within Taiwan's eight-level LTC framework while preserving adequate numbers within each stratum for exploratory analyses. Reasons for not applying for LTC were available as aggregate case-management summaries and were therefore reported descriptively. Individual-level data on non-applicants, post-discharge 30-/90-day readmission or mortality, and formal caregiver burden scales were not available in the analytic dataset.

### Role of discharge-planning nurses

Discharge-planning nurses at NTUH Yunlin Branch serve as specially trained case managers responsible for pre-discharge assessment and transitional-care coordination. Their role includes evaluating functional status using standardized tools (including ADL/IADL and CMS), educating families about LTC options, facilitating applications for government-funded LTC services, and coordinating with physicians, LTC agencies, and families to support safe transitions to the community or care facilities.

### Statistical analysis

Descriptive statistics summarized patient characteristics, discharge outcomes, LTC application, disability levels, and service utilization. Continuous variables are presented as median [interquartile range] and categorical variables as number (%). Comparisons across CMS categories used Kruskal-Wallis tests for continuous variables and chi-square or Fisher's exact tests for categorical variables, as appropriate. All analyses were conducted using IBM SPSS Statistics version 25 (IBM Corp., Armonk, NY, USA).

To strengthen inference beyond univariable comparisons, exploratory multivariable analyses were performed among LTC applicants with complete CMS assessment data (n=75). Because home care and respite care were common outcomes, adjusted relative risks were estimated using modified Poisson regression with robust variance. The number of requested LTC service types (transportation, assistive devices, home care, home-based rehabilitation, respite care, and home modifications) was modeled using Poisson regression with robust variance. Prespecified covariates for primary models were age, sex, and length of stay. ADL and IADL were not entered into the primary CMS-based models because they represent the functional construct underlying disability stratification; instead, separate sensitivity analyses replaced CMS with continuous ADL and IADL scores.

A second sensitivity analysis additionally adjusted for living arrangement and paid caregiver or migrant-worker availability to explore caregiver-context effects. Length of stay across CMS categories was examined using a log-linear regression model adjusted for age and sex. For six prespecified service-specific comparisons, false discovery rate (FDR) adjusted p values were calculated using the Benjamini-Hochberg procedure as a sensitivity check. Given the modest sample size, all multivariable analyses were prespecified as exploratory. A two-sided p value <0.05 was considered statistically significant.

## Results

### Study cohort and LTC application after discharge

A total of 207 cancer patients underwent structured discharge planning in 2024. Discharge outcomes were as follows: 141 patients (68.1%) returned home, 26 (12.6%) were transferred to LTC facilities, 1 (0.5%) was transferred to another hospital, and 39 (18.8%) died during hospitalization. Among the 167 patients discharged home or to care facilities, 75 (44.9%) applied for government-funded LTC services.

Among the 92 patients who did not apply for government-funded LTC, 24 (26.1%) were documented as functionally independent, 16 (17.4%) were ineligible because of age or disability criteria, and 52 (56.5%) relied on family caregiving, privately hired caregiving, or reported a mismatch between available services and their perceived needs (Supplementary Table S1). These aggregate data suggest that non-use reflected not only functional independence but also structural eligibility constraints and alternative caregiving arrangements.

### Characteristics of LTC applicants by CMS category

Among the 75 LTC applicants with complete CMS assessment, 19 (25.3%) had mild disability, 36 (48.0%) moderate disability, and 20 (26.7%) severe disability. Baseline characteristics across CMS categories are summarized in Table 1. Length of stay increased markedly with disability severity, with median stays of 20.0 [9.0-21.5] days in the mild group, 20.5 [13.8-32.5] days in the moderate group, and 45.0 [27.2-74.2] days in the severe group (p<0.001). As expected, ADL and IADL scores declined monotonically across CMS levels (both p<0.001).

The moderate-disability group had the highest median number of requested LTC service types (2.0 [2.0-2.2]) compared with the mild group (1.0 [1.0-2.0]) and severe group (1.0 [1.0-2.2]) (p=0.008), suggesting that service intensity did not increase linearly across disability levels. Patients in the severe-disability group were also more likely to have a paid caregiver or migrant worker than those in the mild group (30.0% vs 0.0%; overall p=0.019), whereas any family caregiver was common across all CMS strata.

### Requested LTC services across CMS categories

Requested service packages differed significantly by CMS category (Table 2). Home care peaked in the moderate-disability group (77.8%) and was less frequent in the severe-disability group (25.0%). In contrast, respite care increased progressively from mild to moderate to severe disability (42.1%, 72.2%, and 100.0%, respectively). The combined request for both home care and respite care was also most common in the moderate-disability group (52.8% vs 21.1% in mild disability and 25.0% in severe disability; raw p=0.028).

After False Discovery Rate (FDR) adjustment across six prespecified service categories, only home care and respite care remained significantly associated with CMS category (FDR-adjusted p=0.002 for both), whereas transportation, assistive devices, home-based rehabilitation, and home modification did not. Overall approval rates were high for most requested services, particularly transportation (95.7%), assistive devices (100.0%), home care (95.5%), and respite care (88.9%) (Supplementary Table S4).

### Multivariable analyses

In the primary multivariable model adjusted for age, sex, and length of stay, moderate disability was associated with a 47% higher number of requested service types than mild disability (aRR 1.47, 95% CI 1.16-1.87; p=0.001), whereas severe disability was not (aRR 1.02, 95% CI 0.75-1.40; p=0.887) (Table 3). For service-specific outcomes, severe disability was strongly associated with respite care (aRR 2.40, 95% CI 1.40-4.09; p=0.001) and inversely associated with home care use (aRR 0.40, 95% CI 0.17-0.95; p=0.038). The likelihood of requesting both home care and respite care was highest in the moderate-disability group (aRR 2.52, 95% CI 0.98-6.47; p=0.054), suggesting a broader combination of community-based services among patients with intermediate disability.

In caregiver-context sensitivity analyses, the association between moderate disability and greater service intensity remained stable (aRR 1.45, 95% CI 1.14-1.83; p=0.002), and the association between severe disability and respite care remained robust (aRR 2.20, 95% CI 1.29-3.74; p=0.004) (Supplementary Table S2). Living alone was associated with greater home care use (aRR 2.01, 95% CI 1.08-3.73; p=0.027), whereas paid caregiver availability was associated with lower home care use (aRR 0.37, 95% CI 0.15-0.93; p=0.034) and borderline greater respite use (aRR 1.19, 95% CI 1.00-1.41; p=0.052).

When CMS was replaced by continuous functional measures, higher IADL scores were associated with home care use (aRR 1.24 per one-point increase, 95% CI 1.06-1.45; p=0.006), whereas lower ADL and lower IADL scores predicted respite care (ADL: aRR 0.94 per 10-point increase, 95% CI 0.89-0.99; p=0.019; IADL: aRR 0.91 per one-point increase, 95% CI 0.84-0.98; p=0.013) (Supplementary Table S3). In the age- and sex-adjusted log-linear model, severe disability was associated with 2.82-fold longer hospitalization than mild disability (95% CI 1.71-4.65; p<0.001), linking CMS-defined disability severity to broader acute-to-community care complexity (Table 3).

## Discussion

This study strengthens the health-services interpretation of CMS-based discharge assessment in post-discharge cancer care. Nearly half of patients discharged home or to care facilities applied for government-funded LTC, underscoring the frequency with which post-acute support needs emerge during cancer care transitions. At the same time, the descriptive data on non-applicants suggest that non-use of government LTC does not simply indicate low need: a substantial proportion of patients were constrained by eligibility rules or substituted family or privately paid caregiving for public services. This observation is consistent with prior literature showing that access to post-discharge supportive care and LTC depends not only on clinical need but also on service design, family resources, and system readiness 11-14.

The most important analytic finding is that CMS captured a non-linear pattern of service need rather than a simple linear increase in utilization with disability severity. Patients with moderate disability had the greatest multi-service intensity and were most likely to request a combined home-care plus respite package, suggesting that partially preserved but unstable function may create the broadest range of transitional-care needs. By contrast, patients with severe disability were characterized less by service diversity and more by near-universal reliance on respite care. Clinically, this pattern is plausible: once dependency becomes extreme, the central unmet need may shift from broader combinations of services toward caregiver support and continuity planning at home 15, 16. Importantly, the direction of these findings was also supported in sensitivity analyses using continuous ADL and IADL scores, which reduces the likelihood that the non-linear pattern was created solely by the chosen CMS groupings.

The strong association between severe CMS status and prolonged hospitalization extends the relevance of CMS beyond LTC referral alone. In this cohort, severe disability was associated with almost threefold longer hospital stay after adjustment for age and sex, indicating that CMS may capture broader care complexity across the acute-to-community continuum. This finding suggests that CMS may help identify patients requiring more complex transition planning and home-care coordination 17, 18. In operational terms, our single-center data generate the hypothesis that CMS could help structure earlier discussions about differentiated post-discharge care bundles—broader community-service combinations for moderate disability and earlier caregiver-support or respite planning for severe disability—but they should not be interpreted as evidence of improved outcomes or implementation readiness without external validation.

Our 2024 cohort should also be interpreted within Taiwan's evolving LTC policy context. During LTC 2.0, hospital discharge-planning programs increasingly linked in-hospital assessment to LTC referral and service planning 9, 10. Since 2026, LTC 3.0 has further emphasized seamless medical-to-LTC transitions and completion of home-care plans before discharge 19. Accordingly, our findings are best viewed as a late-LTC-2.0 snapshot that informs hypotheses about how functional stratification might support post-discharge planning under a more integrated national framework.

The caregiver-context sensitivity analyses provide an additional layer of interpretation. Living alone favored home care, while the presence of a paid caregiver or migrant worker reduced home-care use and showed a borderline association with respite care. Although this dataset did not include a formal caregiver-burden questionnaire, the observed patterns support the idea that caregiver structure shapes the modality of LTC use. This aligns with prior evidence that the availability of informal and paid caregiving influences long-term care utilization and expenditures 11-14, 20, 21.

Several limitations merit emphasis. First, this was a single-center retrospective study with a modest sample size for multivariable modeling, so the adjusted estimates should be interpreted as exploratory. Second, detailed individual-level data were available primarily for LTC applicants, which precluded an adjusted comparison between applicants and non-applicants. The descriptive non-applicant summaries nonetheless suggest that non-use reflected a heterogeneous mixture of low current need, eligibility constraints, alternative family or privately paid caregiving, and potential service mismatch; this group warrants dedicated individual-level and qualitative study. Third, the analytic dataset did not contain post-discharge 30- or 90-day readmission, post-discharge mortality, emergency department use, formal caregiver-burden scales, or cost data. Therefore, any implications regarding healthcare utilization, caregiver outcomes, or resource allocation should be considered hypotheses rather than demonstrated effects. Fourth, the cohort combined solid tumors and hematologic malignancies, but stage, treatment intensity, symptom burden, prognosis, and comorbidity were not available in the analytic model. These unmeasured clinical factors may confound observed associations and limit disease-specific inference. Future work should link hospital discharge data with LTC claims or follow-up registries to compare applicants with non-applicants and to evaluate 30-/90-day readmission, mortality, institutionalization, caregiver-reported burden, cost, and cancer-specific clinical heterogeneity.

Despite these limitations, the present study offers a clinically interpretable framework for strengthening post-discharge cancer care planning. CMS appears useful as a pragmatic way to anticipate which type of community-based service package may need discussion during discharge planning, rather than as a stand-alone tool for resource allocation or practice change. Whether CMS-guided referral improves LTC linkage, readmission, institutionalization, caregiver burden, or cost requires prospective multicenter validation.

## Conclusion

This study demonstrates that CMS-based assessment at hospital discharge can identify meaningful heterogeneity in LTC needs among cancer patients eligible for post-discharge LTC consideration. Moderate disability was associated with the highest multi-service intensity, whereas severe disability was characterized by dependence on respite care and substantially longer hospitalization.

These findings suggest that CMS is most informative not as a simple severity label, but as a pragmatic framework for anticipating different service packages during post-discharge cancer care transitions. The next step for a higher-impact evaluation is to validate these patterns in larger multicenter datasets that include individual-level non-applicant comparisons, downstream outcomes, caregiver burden, cost, and cancer-specific clinical variables.

## Supplementary Material

Supplementary tables.

## Figures and Tables

**Table 1:** Characteristics of LTC applicants with complete CMS assessment according to CMS category

Variable	Mild disability (CMS 2-3, n=19)	Moderate disability (CMS 4-6, n=36)	Severe disability (CMS 7-8, n=20)	P value
Age, years	72.0 [66.5-75.0]	73.0 [69.0-78.8]	78.0 [68.5-83.2]	0.189
Length of stay, days	20.0 [9.0-21.5]	20.5 [13.8-32.5]	45.0 [27.2-74.2]	<0.001
ADL score	95.0 [75.0-100.0]	60.0 [50.0-70.0]	22.5 [8.8-35.0]	<0.001
IADL score	5.0 [4.0-5.5]	4.0 [4.0-4.0]	0.0 [0.0-1.0]	<0.001
No. of requested LTC service types	1.0 [1.0-2.0]	2.0 [2.0-2.2]	1.0 [1.0-2.2]	0.008
Female	5 (26.3%)	14 (38.9%)	5 (25.0%)	0.468
Living alone	2 (10.5%)	3 (8.3%)	0 (0.0%)	0.360
Disability certificate	2 (10.5%)	7 (19.4%)	5 (25.0%)	0.504
History of falls	3 (15.8%)	6 (16.7%)	4 (20.0%)	0.931
Married	11 (57.9%)	23 (63.9%)	13 (65.0%)	0.881
Paid caregiver / migrant worker	0 (0.0%)	4 (11.1%)	6 (30.0%)	0.019
Any family caregiver	13 (68.4%)	30 (83.3%)	19 (95.0%)	0.090
Self as caregiver	11 (57.9%)	21 (58.3%)	6 (30.0%)	0.097

Values are median [IQR] or n (%). P values are from Kruskal-Wallis or chi-square tests, as appropriate.

**Table 2:** Requested LTC services according to CMS category

Requested LTC service	Mild disability (CMS 2-3, n=19)	Moderate disability (CMS 4-6, n=36)	Severe disability (CMS 7-8, n=20)	Raw P value	FDR-adjusted P value
Home care	11 (57.9%)	28 (77.8%)	5 (25.0%)	<0.001	0.002
Respite care	8 (42.1%)	26 (72.2%)	20 (100.0%)	<0.001	0.002
Transportation	4 (21.1%)	13 (36.1%)	6 (30.0%)	0.514	0.566
Assistive devices	4 (21.1%)	7 (19.4%)	0 (0.0%)	0.095	0.142
Home-based rehabilitation	0 (0.0%)	1 (2.8%)	3 (15.0%)	0.073	0.142
Barrier-free home modification	1 (5.3%)	2 (5.6%)	0 (0.0%)	0.566	0.566
Home care + respite (exploratory)	4 (21.1%)	19 (52.8%)	5 (25.0%)	0.028	—

FDR-adjusted p values were calculated for the six prespecified service-specific comparisons using the Benjamini-Hochberg procedure. The combined home care + respite outcome was exploratory and was not included in the FDR family

**Table 3:** Primary multivariable models for service utilization and length of stay

Outcome	Term	Adjusted estimate (95% CI)	P value
Number of requested service types	Moderate vs mild CMS	1.47 (1.16-1.87)	0.001
Number of requested service types	Severe vs mild CMS	1.02 (0.75-1.40)	0.887
Number of requested service types	Age (per 10 years)	0.88 (0.79-0.99)	0.031
Number of requested service types	Female sex	0.90 (0.73-1.11)	0.319
Number of requested service types	Length of stay (per 10 days)	1.04 (1.00-1.09)	0.032
Home care	Moderate vs mild CMS	1.36 (0.88-2.11)	0.171
Home care	Severe vs mild CMS	0.40 (0.17-0.95)	0.038
Home care	Age (per 10 years)	0.82 (0.64-1.06)	0.130
Home care	Female sex	1.11 (0.80-1.55)	0.522
Home care	Length of stay (per 10 days)	1.04 (0.94-1.13)	0.457
Respite care	Moderate vs mild CMS	1.73 (0.98-3.05)	0.057
Respite care	Severe vs mild CMS	2.40 (1.40-4.09)	0.001
Respite care	Age (per 10 years)	1.02 (0.93-1.13)	0.624
Respite care	Female sex	0.90 (0.67-1.21)	0.483
Respite care	Length of stay (per 10 days)	0.99 (0.97-1.02)	0.696
Home care + respite	Moderate vs mild CMS	2.52 (0.98-6.47)	0.054
Home care + respite	Severe vs mild CMS	1.11 (0.33-3.75)	0.863
Home care + respite	Age (per 10 years)	0.88 (0.62-1.27)	0.497
Home care + respite	Female sex	1.05 (0.57-1.92)	0.874
Home care + respite	Length of stay (per 10 days)	1.03 (0.91-1.16)	0.641
Length of stay	Moderate vs mild CMS	1.37 (0.89-2.13)	0.156
Length of stay	Severe vs mild CMS	2.82 (1.71-4.65)	<0.001
Length of stay	Age (per 10 years)	1.09 (0.88-1.35)	0.441
Length of stay	Female sex	1.14 (0.79-1.65)	0.495

Adjusted relative risks (aRRs) are shown for binary outcomes and the number of requested service types. Length of stay was modeled using log-linear regression and is presented as a ratio. Primary models were adjusted for age, sex, and length of stay, except the length-of-stay model, which was adjusted for age and sex.

## Data Availability

All data analyzed in this revised manuscript are presented in the main text, tables, or supplementary tables.

## Abbreviations

ADL: Activities of Daily Living
AJCC: American Joint Committee on Cancer
CMS: Case-Mix System
EMR: Electronic Medical Record
IADL: Instrumental Activities of Daily Living
LTC: Long-Term Care
UICC: Union for International Cancer Control
