# Supplementary material for: Functional Stratification and Long-Term Care Service Patterns among Discharged Patients with Cancer: A Retrospective Study from Taiwan
Source: J Cancer. 2026 May 11;17(5):1032–8. doi: 10.7150/jca.134200 (PMC13189816; doi:10.7150/jca.134200)
Supplement: Supplementary file 1 — Supplementary tables. [file jcav17p1032s1.pdf]

Supplementary Table S1. Aggregate reasons for not applying for government-funded LTC among non-applicants

Reason for not applying for LTC (n=92)	n	%
Independent in ADL / no current LTC need	24	26.1%
Ineligible by age/disability criteria	16	17.4%
Family care, privately hired caregiver, or perceived service mismatch	52	56.5%

Only aggregate descriptive data were available for non-applicants; individual-level covariates were not available for adjusted applicant-versus-non-applicant comparisons.

Supplementary Table S2. Sensitivity models additionally adjusted for living arrangement and paid caregiver / migrant worker availability

Outcome	Term	Adjusted estimate (95% CI)	P value
Number of requested service types	Moderate vs mild CMS	1.45 (1.14–1.83)	0.002
Number of requested service types	Severe vs mild CMS	0.96 (0.68–1.35)	0.807
Number of requested service types	Age (per 10 years)	0.88 (0.78–0.98)	0.019
Number of requested service types	Female sex	0.90 (0.72–1.12)	0.326
Number of requested service types	Length of stay (per 10 days)	1.04 (1.00–1.08)	0.049
Number of requested service types	Living alone	0.78 (0.45–1.34)	0.363
Number of requested service types	Paid caregiver / migrant worker	1.22 (0.91–1.63)	0.193
Home care	Moderate vs mild CMS	1.52 (0.94–2.47)	0.090
Home care	Severe vs mild CMS	0.51 (0.21–1.22)	0.129
Home care	Age (per 10 years)	0.84 (0.66–1.07)	0.161
Home care	Female sex	1.13 (0.80–1.58)	0.495
Home care	Length of stay (per 10 days)	1.06 (0.96–1.17)	0.248
Home care	Living alone	2.01 (1.08–3.73)	0.027
Home care	Paid caregiver / migrant worker	0.37 (0.15–0.93)	0.034
Respite care	Moderate vs mild CMS	1.70 (0.98–2.96)	0.060
Respite care	Severe vs mild CMS	2.20 (1.29–3.74)	0.004
Respite care	Age (per 10 years)	1.01 (0.92–1.11)	0.806
Respite care	Female sex	0.89 (0.66–1.20)	0.435
Respite care	Length of stay (per 10 days)	0.99 (0.97–1.02)	0.562
Respite care	Living alone	0.57 (0.22–1.45)	0.237

Outcome	Term	Adjusted estimate (95% CI)	P value
Respite care	Paid caregiver / migrant worker	1.19 (1.00–1.41)	0.052

Models additionally adjusted for living alone and paid caregiver / migrant-worker availability, together with age, sex, and length of stay.

Supplementary Table S3. Sensitivity models replacing CMS with continuous ADL and IADL scores

Outcome	Term	Adjusted estimate (95% CI)	P value
Home care	ADL (per 10-point increase)	0.99 (0.91–1.07)	0.814
Home care	IADL (per 1-point increase)	1.24 (1.06–1.45)	0.006
Home care	Age (per 10 years)	0.87 (0.68–1.13)	0.304
Home care	Female sex	1.14 (0.80–1.62)	0.464
Home care	Length of stay (per 10 days)	1.03 (0.94–1.13)	0.514
Respite care	ADL (per 10-point increase)	0.94 (0.89–0.99)	0.019
Respite care	IADL (per 1-point increase)	0.91 (0.84–0.98)	0.013
Respite care	Age (per 10 years)	0.99 (0.89–1.10)	0.852
Respite care	Female sex	0.95 (0.71–1.28)	0.747
Respite care	Length of stay (per 10 days)	0.98 (0.94–1.01)	0.194
Number of requested service types	ADL (per 10-point increase)	0.98 (0.94–1.02)	0.354
Number of requested service types	IADL (per 1-point increase)	1.06 (0.99–1.13)	0.112
Number of requested service types	Age (per 10 years)	0.90 (0.80–1.02)	0.097
Number of requested service types	Female sex	0.94 (0.76–1.16)	0.562
Number of requested service types	Length of stay (per 10 days)	1.04 (1.00–1.08)	0.046

These models replaced CMS category with continuous ADL and IADL scores while retaining age, sex, and length of stay.

Supplementary Table S4. Approval rates of requested LTC services among applicants

Service	Requested (n)	Approved (n)	Approval rate
Transportation	23	22	95.7%
Assistive devices	11	11	100.0%
Home care	44	42	95.5%
Home-based rehabilitation	4	3	75.0%
Respite care	54	48	88.9%
Barrier-free home modification	3	2	66.7%

Approval status reflects the service-authorization outcome recorded in the available LTC application dataset.
